# A Novel Crowdsourcing Model for Micro-Mobility Ride-Sharing Systems

**DOI:** 10.3390/s21144636

**Published:** 2021-07-06

**Authors:** Mohammed Elhenawy, Mostafizur R. Komol, Mahmoud Masoud, Shi Qiang Liu, Huthaifa I. Ashqar, Mohammed Hamad Almannaa, Hesham A. Rakha, Andry Rakotonirainy

**Affiliations:** 1 Centre for Accident Research and Road Safety, Queensland University of Technology, Brisbane 4059, Australia; mohammed.elhenawy@qut.edu.au (M.E.); mdmostafizurrahman.komol@hdr.qut.edu.au (M.R.K.); mahmoud.masoud@qut.edu.au (M.M.); r.andry@qut.edu.au (A.R.); 2 School of Economics and Management, Fuzhou University, Fuzhou 350108, China; 3 Booz Allen Hamilton, Washington, DC 20003, USA; hiashqar@vt.edu; 4 Civil Engineering Department, King Saud University, Riyadh 11362, Saudi Arabia; malmannaa@ksu.edu.sa; 5 Engineering and Director of the Center for Sustainable Mobility, Virginia Tech Transportation Institute, Blacksburg, Virginia 24060, Australia; hrakha@vt.edu

**Keywords:** micro-mobility, ride-sharing, agent-based modelling, crowdsourcing

## Abstract

Substantial research is required to ensure that micro-mobility ride sharing provides a better fulfilment of user needs. This study proposes a novel crowdsourcing model for the ride-sharing system where light vehicles such as scooters and bikes are crowdsourced. The proposed model is expected to solve the problem of charging and maintaining a large number of light vehicles where these efforts will be the responsibility of the crowd of suppliers. The proposed model consists of three entities: suppliers, customers, and a management party responsible for receiving, renting, booking, and demand matching with offered resources. It can allow suppliers to define the location of their private e-scooters/e-bikes and the period of time they are available for rent. Using a dataset of over 9 million e-scooter trips in Austin, Texas, we ran an agent-based simulation six times using three maximum battery ranges (i.e., 35, 45, and 60 km) and different numbers of e-scooters (e.g., 50 and 100) at each origin. Computational results show that the proposed model is promising and might be advantageous to shift the charging and maintenance efforts to a crowd of suppliers.

## 1. Introduction

At present, micro-mobility is a promising urban mobility solution [1]. The term “micro-mobility” deals with the incorporation of a short trip by a small vehicle operation. In micro-mobility, transportation mobility is constrained to a very limited range of trips for light vehicles only [2]. Vehicles of light categories, such as bicycles, motorbikes, electric bikes (e-bikes), electric scooters (e-scooters), shared bicycles, and some riding devices like skateboards are considered micro-mobility rides [3]. Micro-mobility ride sharing is a useful system that provides sustainability for the ever-growing population, traffic congestion, and greenhouse impact [4]. Environmental pollution and emissions can be minimized to an enormous amount by utilizing micro-mobility rather than regular motor vehicles [5,6]. Public interest in micro-mobility like bike-sharing systems (BSS) or scooter-sharing systems (SSS) is recently increasing with the rapid transformation of the transportation system. This strategy gives an affordable and rapid passage opportunity, especially for traffic congested routes, thereby saving commuters from long waiting time [7,8,9].

The micro-mobility ride-sharing system is categorized to belong to the fourth generation [10], where dock-based systems are considered as the latest updated versions of the first two generations [11,12]. The emergence of fourth-generation dockless ride-sharing has revolutionized the micro-mobility ride-sharing market and [13] due to mass public deliberation to the sharing economy, sustainability, and health transports [14]. The early invention of the micro-mobility ride-sharing strategy was started as dockless bike-sharing, but this system was not prioritized enough due to the lack of technologies that could fulfil the system’s drawbacks. Instead, people accepted dock-based ride-sharing as a more flexible and reliable approach compared to dockless ride-sharing. However, in these modern days, new technologies have assured that the dockless ride-sharing is more flexible and reliable in terms of users’ expectation [15]. Moreover, this system is handy on installation and less intricate for the smartcard integration system on transit and facilitated with advanced power-assisting features [10]. The dock-based system causes an extra infrastructure cost to be paid for the agglomeration and locking security of micro-mobility rides [15]. Nonetheless, the dockless system is more flexible than the dock-based system. Their advantages and superiority of implementation vary based on the feature of locations [16] and on the purpose of the trip, how fast the user walks, the destination, budgets for the rides, reliability value, and any other operator attributes [17]. Even the dockless micro-mobility system is bound to many limitations. Many of such businesses quickly collapsed due to the hardship in maintaining sustainability, oversized fleets, and vandalism [15,18,19]. Moreover, in the dockless system, the range of the distance to be traveled by scooters must be restricted due to the limitation of the battery power charge. Commuters will resort to abandoning the shared scooter halfway to their destination if the charge drops below the usability condition [16]. In some European countries, the usage of private e-scooters is rapidly overgrowing [20]. The extensive use of micro-mobility rides on public roads often causes annoyance and conflict with the safety of regular pedestrians [21]. Therefore, a profusion of complaints about the exaggeration of light vehicles, which negatively affects the business idea of micro-mobility ride-sharing exists. In this study, we proposed a new model for the micro-mobility ride-sharing systems, where e-scooters and bikes are crowdsourced. This novel model consists of three entities: the suppliers, the customers, and the management party. The proposed model is believed to offer several advantageous features for all classes included in this model.

The clarification of the ambiguity of previous inventions and the evaluation of novel ideas on the micro-mobility ride-sharing system are pivotal research topics to focus on in the modern era. Considering all the challenges and hurdling experiences with conventional micro-mobility ride-sharing systems, improvement and modifications are desirable to cover the drawbacks of this field. Further research efforts are required to give the micro-mobility ride-sharing system a better fulfilment of user expectations. The innovation models like the micro-mobility ride-sharing are getting popular concern and will be welcomed in public appearance if they can fulfil the challenges confronting dock-based and dockless ride-sharing systems. This study presents a novel model for the micro-mobility ride-sharing system, focusing on removing the recharging and maintenance burden from the operator. The proposed model offers advantageous features for all classes included in this business strategy.

The rest of the paper is outlined as follows. The literature review on the micro-mobility ride-sharing system is presented in Section 2. A novel crowdsourcing model is presented in Section 3, where Section 4 presented experimental results. Finally, the discussion and conclusion are presented in Section 5.

## 2. Literature Review

Sharing micro-mobility rides for public necessity, and business expansion is not an unprecedented scheme. This idea first evolved around 1965, but for the lack of technologies to track down customers, users used to remain anonymous, and miserable incidents like stealing and destroying of bikes frequently occurred, which led the system to collapse for practical implementation [22,23,24]. As technology arises with smartphones and GPS systems, customers have become able to easily track and locate the nearest available sharable rides through GPS and even unlock them using their smartphones [16,25]. This system is spreading to a greater extent from the last decades with the advancement of relevant technologies and management policies [26]. Therefore, researchers are inspired to take immense efforts to improve micro-mobility ride-sharing management and technologies considering that it is a popular concern and is being used ubiquitously.

The micro-mobility ride-sharing system is being developed through time and modified with both technology and policy improvements. An innovative simulation study of models without redistribution and with a simple redistribution is proposed for a more effectual bike redistribution system [27]. When customers intend to rent a bike, they need to know the rental information exploitation mechanisms about the bike pick-up and return. This helps in fulfilling their demand properly and prevents probable queue making during renting. In another research, machine learning-based univariate and multivariate regression algorithms have been implemented to model available bikes in rental stations [28,29,30]. The greedy randomized adaptive search procedure algorithm has been utilized and improved for the bicycle rebalancing problem to solve the unavailability of bicycles to rent [31]. Random forest and multi-layer perception have been used to predict bike availability in different rental stations using real-world data [32]. Here, the bike pick-up and return are optimized to maintain balance for bike availability based on the prediction [33]. Nowadays, mobile phone apps are being initiated and developed to locate nearby stations for rent and ride availability. Route planning and possible parking for commuters are also being presented through these apps [34]. Attempts to solve the ride unavailability in rental stations have also been made using policymaking. The characteristics of customers in renting rides from different stations have been evaluated. Moreover, the tendency of choosing an alternative station with fewer available bikes has been found among customers than preferred ones [35]. Dockless ride-sharing is getting superior with economic advantages in the bike-sharing market by implementing a remote mobile payment system and the emergence of big-data computation [8,15,36,37,38]. However, the redistribution due to the imbalance of bike pick-up and return is more challenging for the dockless ride-sharing system. Maintaining a win–win situation for customers by providing monetary incentives in renting rides is highly effective in balancing the dockless ride-sharing system redistribution [39].

E-scooter share trip trajectories are provided at the street link level with a precise construction of the trajectory trip inventories [40]. The contribution of character individuals to the reduction of the heat alert through the bike-sharing system has been analysed in research, where the factors of age and gender show different behaviours. Different weather and environmental conditions and the ability to expand the system [41] have also been found responsible for the variance of micro-mobility ride hiring by users [42,43]. Apart from age and gender, the trip purpose, time of the day, day of the week, population density, median household income, and some other demographic and external factors have been found as a reagent in other studies on Washington DC and Austin, TX for the ride-sharing variability [44,45]. To understand people’s perceptions and opinions over micro-mobility ride-sharing, researchers accumulated data from social media platforms like Twitter and Facebook and utilized the machine learning approach to evaluate the current toleration and support of the ride-sharing business at present [46]. The complexity and the sophistication of mobile applications that operate in micro-mobility ride-sharing assistance are the influential factors behind the use of ride-sharing. The young generation is highly acquainted with complex apps and has easily been driven to use micro-mobility because they properly understand the feature and facilities of the system through the apps.

The micro-mobility ride-sharing system is being gradually developed and needs future improvements and modifications to solve myriads of challenges that people face every day in operating this business practice. This study proposes a new model for the micro-mobility ride-sharing system, where light vehicles, such as electric scooters and bikes, are crowdsourced. An advantage of this new model is that it shifts the charging and maintenance efforts to the crowd of suppliers. The maintenance and charging costs due to the high repositioning rates of shared scooters are much higher in a conventional ride-sharing system [16], and these costs often become challenging to customers or a business party. In our proposed strategy, this cost will be covered by the suppliers, and the management party will be relieved from the burden of this cost. However, the incentive payments by the customers to suppliers will cover some of its expenses as a shared policy.

## 3. Novel Crowdsourcing Model

The proposed model consists of three entities:

- suppliers (i.e., people offering their private e-scooters/e-bikes (Throughout this paper, we occasionally use only the term “e-scooter(s)” to refer to all other micro-mobility vehicles because e-scooters are the main vehicles used in the tested dataset in this study.) for hire);
- customers (i.e., people creating the hiring demand); and
- a management party (i.e., responsible for receiving hiring booking demands and matching them with the offered resources).

It allows the suppliers to define the location of their private e-scooter/e-bike and the period of time they are available for rent. The model will allow suppliers to have their e-scooters/e-bikes hired and returned at the end of the renting period to the same location or another near location. In other words, the management party needs to match the e-scooter/e-bike to a series of hiring demands with the last demand as a destination very close to the initial location of the e-scooter/e-bike at the start of the hiring period.

An advantage of this new model is that it shifts the charging and maintenance efforts to the crowd of suppliers. The maintenance and charging costs in a conventional ride-sharing system are much higher due to the high repositioning rates of shared scooters [16], and this often becomes challenging to customers or business parties. In our proposed strategy, the suppliers will cover this cost, and both the customers and the maintenance party will be relieved from the burden of sharing this cost. The incentive payments by the customers to the suppliers will cover some of its expenses as a shared policy.

Several approaches could be used to match the supply and demand in a ride-sharing system. We can choose the matching approach depending on the booking scheme allowed by the model. The first possible approach is a data-driven one suitable for real-time booking. The most naive variant of the data-driven approach is what can be referred to as the two-leg round-trip approach. The current e-scooter service is assumed to be meeting only 50% of the total demand volume; thus, we used the available data about the current trips to estimate the origin–destination matrix of the non-observed/hidden demand. Consequently, we plan routes such that we assign e-scooters located at route endpoints to service this route. The e-scooters will be assigned trips only over this route such that it travels back and forth between the two ends of the route, and the destination of the last trip is the e-scooters’ location at the start of the day. This will require the management application to ask renters to enter the origin and destination of the trip to find them the e-scooter assigned to this route. Moreover, the management should not allow e-scooters to get out of their assigned routes. In this approach, the planned routes may vary based on the day-of-the-weak and the hour of the day to meet the expected demand. These route variations make the model flexible and capable of meeting the demand in a more efficient manner. Allowing more than two-leg round-trips is more complicated than a data-driven approach. We use the estimated O–D matrix to establish routes between more than two points such that an e-scooter can freely move between this subset of points during its renting period. However, at the end of the rental period, the final destination of the e-scooter should be as close as possible to its initial location. We will use herein an agent-based modelling (ABM) approach to establish simple management rules suitable for the two-leg round-trip approach.

### 3.1. Proposed Agent-Based Modelling

This subsection discusses the ABM framework of the proposed new model. In Agent-based modelling (ABM) we define the agent’s behaviour as a set of simple rules. ABM is a computational modelling framework that enables agents to execute their rules and interact with other agents in a complex system. By observing and aggregating the outcome of the interactions between the agent we can understand how the complex system is behaving without trying to describe the complex system through mathematical modelling. In general, ABM provides an in-silico lab, where we can test and understanding complex systems by defining simple rules at the level of individual agents.

In the proposed model, we established a set of simple rules that will yield a suitable behaviour of the proposed e-scooter model that has three types of agents:

- e-scooter agent,
- central agent, and
- demand generator agent.

The e-scooter agent has four state variables, namely home location, current location, availability, and current battery range. The other two agents do not have state variables. The demand generator has one behaviour rule. Every second, the demand generator looks at the trip’s data and informs all e-scooter agents of new demand requests, including the trip information. In other words, if there are concurrent demands, the demand generator agent chooses one of them to broadcast and wait until the demand is processed. It then broadcasts another to the concurrent demand requests. The e-scooter agent rule checks if its current location is the same as the trip origin, its battery level, and the availability of e-scooters. If all of these conditions are satisfied, then the e-scooter agent sends an expression of interest (EOI) to the central agent. The central agent then receives EIOs to serve a particular demand from all e-scooter agents and chooses the winner based on the system’s own rules. In this experimental work, we propose five rules to choose the winner as follows:

- The first rule chooses an e-scooter agent randomly from a subset of e-scooter agents submitted to the EOI.
- The second rule chooses the e-scooter agent that submitted the EOI and has the largest remaining charge in its battery.
- The third rule randomly chooses an e-scooter agent from a subset of e-scooter agents, which submitted the EOI, whose destination of the demand (i.e., trip) is the home location of the e-scooter agent.
- The fourth rule chooses the e-scooter agent, which submitted the EOI, whose destination of the demand (i.e., trip) is the home location of the e-scooter agent and has the largest remaining charge in its battery.
- The fifth rule drops the demand if there is no EOI.

### 3.2. Data Analysis and Statistical Methodology

This study used two datasets from Austin, Texas and Chicago, Illinois. In the following subsections, we will briefly describe the two datasets.

#### 3.2.1. Austin Dataset

This study used a dataset collected from Austin, Texas and is publicly available from the City of Austin [47]. The dataset contains approximately 9.2 million trips taken by users for either e-bikes or e-scooters from December 2018 to January 2020. Each trip is represented in a row with 18 features each: trip ID, device ID, vehicle type (e-scooter or e-bike), trip duration (in seconds), trip distance (in meter), start time of the trip, end time of the trip, month, hour, day of week, year, council district (both start and end), and census tract (both start and end). Table 1 shows the total number of e-scooter and e-bikes that operate in the city.

**Figure 1:**
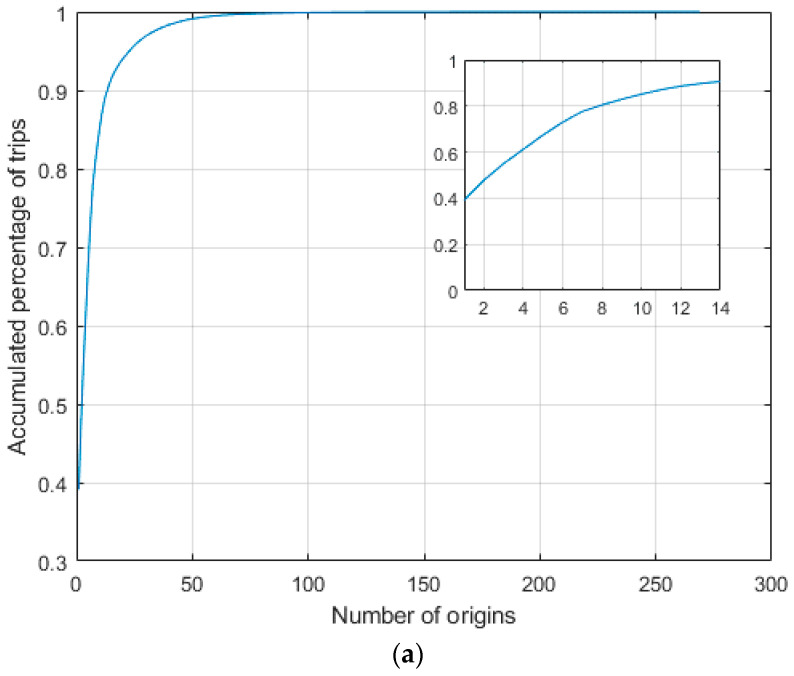

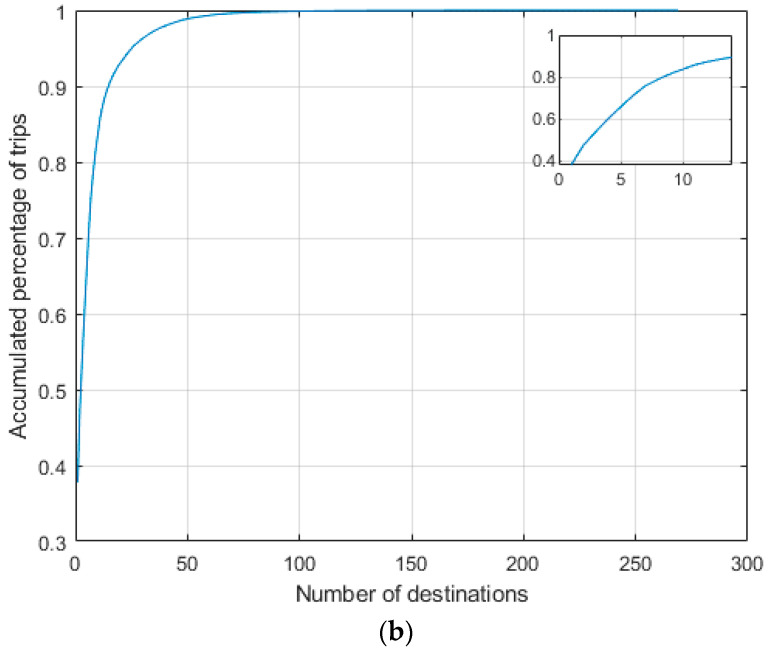

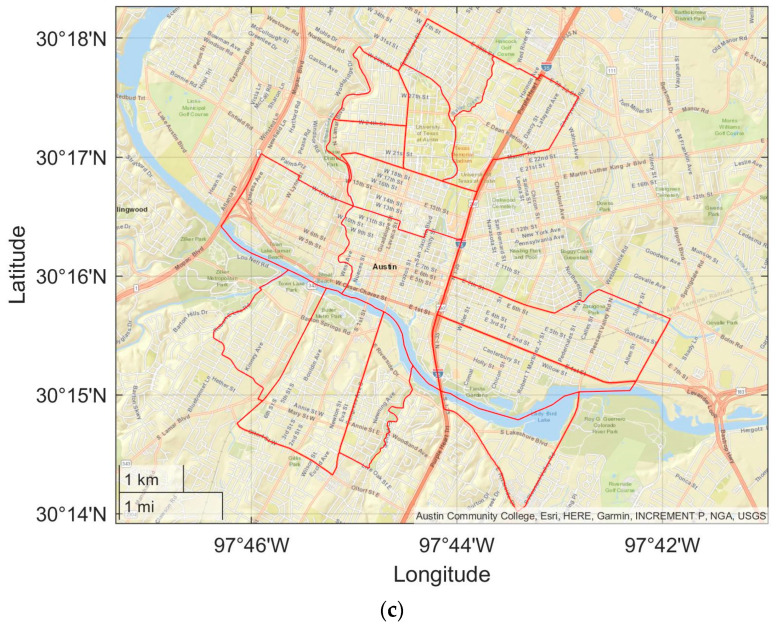

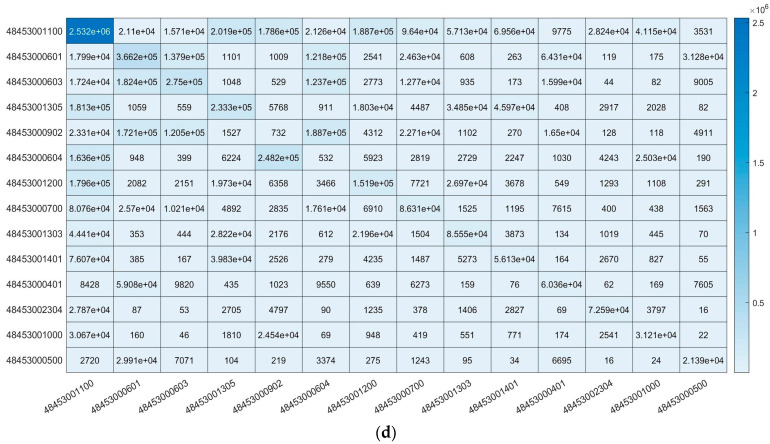
Most of the trips were concentrated in contiguous census tracts: (**a**) accumulated percentage of trips versus number of origins; (**b**) accumulated percentage of trips versus number of destinations; (**c**) 14 census tract that include approximately 90% of the total trips; and (**d**) heat map of the O–D matrix of the 14 census tracts.

It is noted that we used the census tracts of the origin and the destination of each trip to estimate the origin–destination (O–D) matrix. We removed all the trips missing the start census tract and/or end census tract before constructing the origin–destination (O–D) matrix. The number of trips after removing the abovementioned trips reached 9,174,541 trips, which was originally 9,231,107 trips. We had two observations based on the O–D matrix: first, most of the trips were concentrated between particular census tracts; and second, a significant percentage of the trips started and ended in the same census tract as shown in Figure 1. Both observations were expected because the majority of the trips were e-scooter trips that were usually short trips, and e-scooters were deployed in a limited area in the Austin downtown area. To this end, in the following analysis, we used a reduced O–D by selecting 14 census tracts shown in Figure 1c,d, which included almost 90% of the total trips in Austin, TX. The large numbers on the diagonal of the matrix in Figure 1d mean that a high percentage of the trips start and end in the same census tract.

#### 3.2.2. Chicago Dataset

This dataset was collected at the city of Chicago during the 2020 e-scooter pilot. Bird, Lime and Spin participated in this pilot. The data covers four months from mid-August to mid-December. E-scooters were allowed to operate from 5 a.m. to 10 p.m. The dataset consists of 631k trips. Each trip is represented in a row with 16 features each: trip ID, start time, end time, trip duration (in seconds), trip distance (in meter), vendor/operator, start community area number, end community area number, start community area name, end community area name, start centroid latitude, start centroid longitude, start centroid location, end centroid latitude, end centroid longitude and end centroid location.

## 4. Experimental Results

### 4.1. Austin Dataset

In this section, we used the trips’ data of the reduced O–D matrix to investigate the concept of the new model. We used a real dataset of the reduced O–D matrix to run our proposed ABM for different combinations of the central agent rules and chose the combination that satisfies our target performance. Furthermore, we employed two criteria to evaluate the aggregated behaviour of the ABM when changing the central agent rules. The first criterion is called the home index (HI), which is defined as the percentage of the e-scooter agents that ended up at the users’ home location at the end of the operation hours:

(1) $$HI\left(\%\right)=\frac{\sum_{i=1}^{N}I_{e-\mathrm{scooter}\;i\;\mathrm{agents}\;\mathrm{that}\;\mathrm{ended}\;\mathrm{up}\;\mathrm{at}\;\mathrm{the}\;{\mathrm{users}}^{’}\;\mathrm{home}\;\mathrm{locations}\;}}{N}$$

where, I is an indicator function that equals one when is an e-scooter that ended up at a user’s home at the end of the operation hours, and N is number of e-scooters that satisfied at least one demand during the operation hours. The second criterion is the percentage of satisfied demand (PSD), which is the ratio of the demand/trip met at least once during the operation hours over the total number of trips.

(2) $$PSD\left(\%\right)=\frac{number\;of\;satisfied\;trips\;}{total\;number\;of\;trips}$$

We ran the proposed ABM simulation using 389 days’ worth of data. We assumed that all e-scooters had the same maximum battery range in the same run and that the operation hours started with the same number of e-scooters at each of the 14 origins for each run. We simulated the proposed ABM six times using a maximum battery range of (35, 45, and 60 km) and different numbers of e-scooters at each origin (50 and 100). The HI and the PSD were calculated every day at different combinations of the proposed centre agent rules. We then compared the result using a mixed-effect Gamma regression model to explain the variability in the HI and the PSD in terms of the combination of the proposed rules. Table 2 shows the indicator variables used to encode the tested combination.

**Figure 2:**
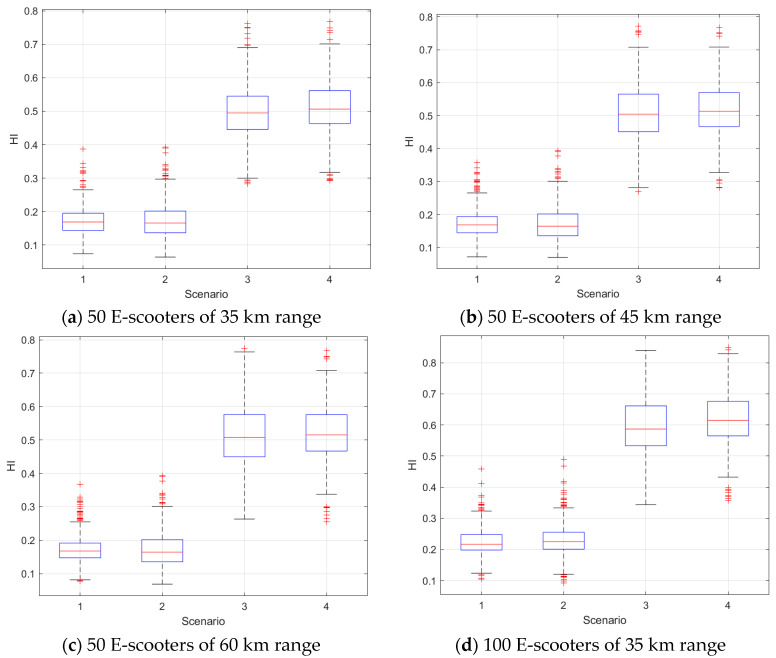

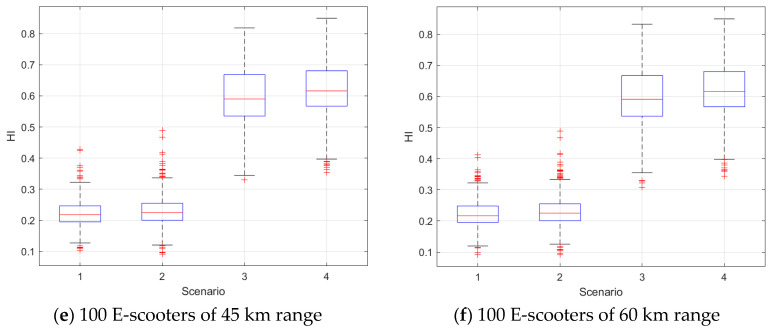
HI results at different scenarios, number of e-scooters at each census tract, and battery ranges.

The e-scooter trips for 389 days were used in the ABM environment to simulate the proposed model and estimate the HI for the 389 days. Figure 2 shows box plots of the HI results versus different scenarios. Scenarios 3 and 4 yielded a higher HI than the other two scenarios because they included the rule of which destination of the demand (i.e., trip) is the home location of the e-scooter agent.

We also used the Gamma mixed-effect model to test whether the differences in the HI results between the scenarios were statistically significant. Table 3 presents that the indicator variables corresponding to Scenarios 3 ($X_{2}$) and 4 ($X_{3}$) were significant. On the contrary, the indicator variable for Scenario 2 was significant only when the system contained 100 e-scooters. The HI results implied that when the system contained 50 e-scooters, the HI of Scenarios 3 and 4 was significantly larger than that of Scenario 1. At the same condition of 50 e-scooters, the HI of Scenario 2 was not statistically significant. Finally, we found that at 100 e-scooters, the HI of Scenarios 2–4 was significantly higher than that of Scenario 1.

**Figure 3:**
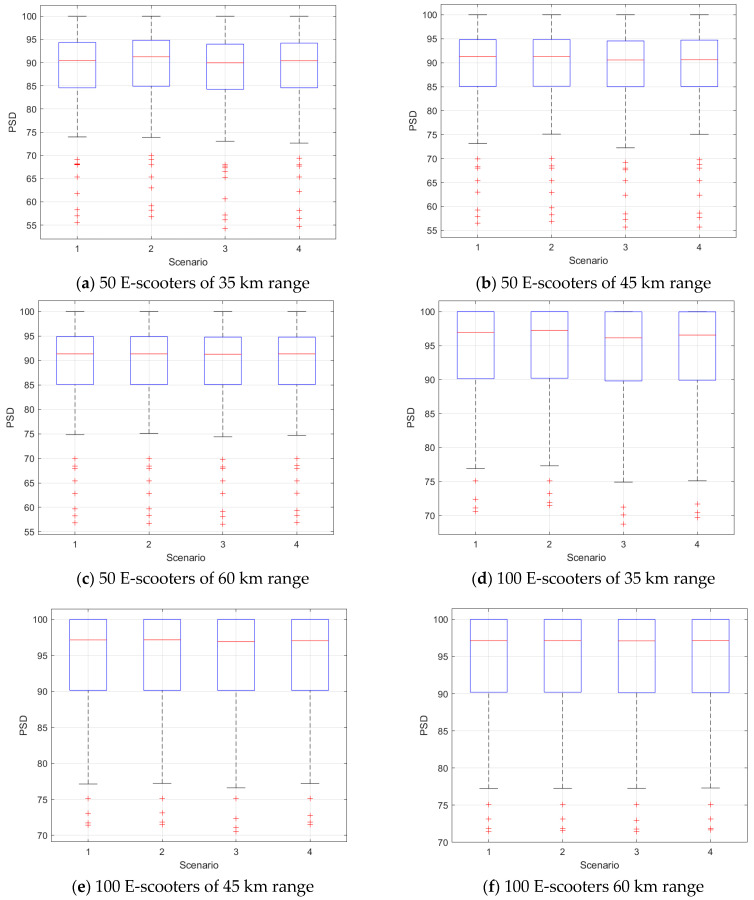
PSD results at different scenarios, number of e-scooters at each census tract, and battery ranges.

We next compared the four scenarios in terms of the PSD at different conditions of the e-scooter number and battery ranges. Figure 3 shows box plots of the PSD versus the scenarios. Almost all scenarios had the same PSD values at the same conditions, and the PSD increased as the used number of e-scooters in the model increased at each census tract. We also modelled the PSD results using a mixed-effect Gamma regression model as a function in the scenarios. Table 4 presents the *p*-values of the indicator variables corresponding to the scenarios. These *p*-values dictated that Scenario 3 was different from Scenario 1 at all conditions.

### 4.2. Chicago Dataset

For the sake of completeness and to prove that the proposed crowdsourcing model can be applied at any city/geographical area, we applied the ABM framework to control the proposed model using the Chicago dataset. We evaluated the proposed model at different battery ranges, different numbers of e-scooters and different four scenarios. The O-D matrix of this data is bigger where we have 77 different community areas used as the origins and destinations of the trips.

**Figure 4:**
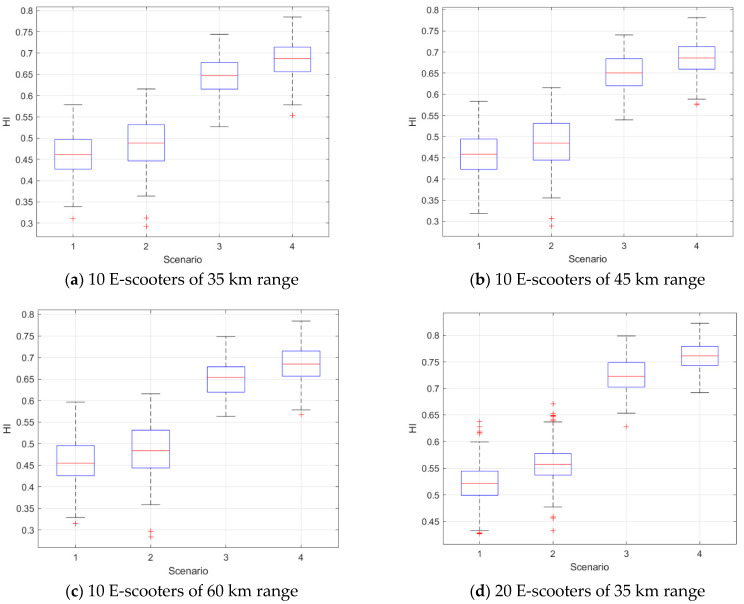

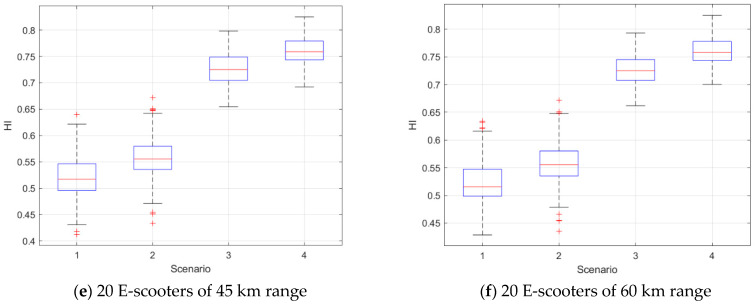
HI results at different scenarios, number of e-scooters at each census tract, and battery ranges.

Figure 4 shows box plots of the HI results versus different scenarios. We obtained the same trend as in the previous dataset where Scenarios 3 and 4 yielded a higher HI than the other two scenarios. This result confirms that the rule of which destination of the demand (i.e., trip) is the home location of the e-scooter agent is important to grantee a good percentage of the e-scooters end at their home located at the end of the operation hours.

Gamma mixed-effect model is used to test whether the differences in the HI results between the scenarios were statistically significant. As shown in Table 5, Scenarios 2–4 have a statistically significant larger HI compared to the first scenario.

**Figure 5:**
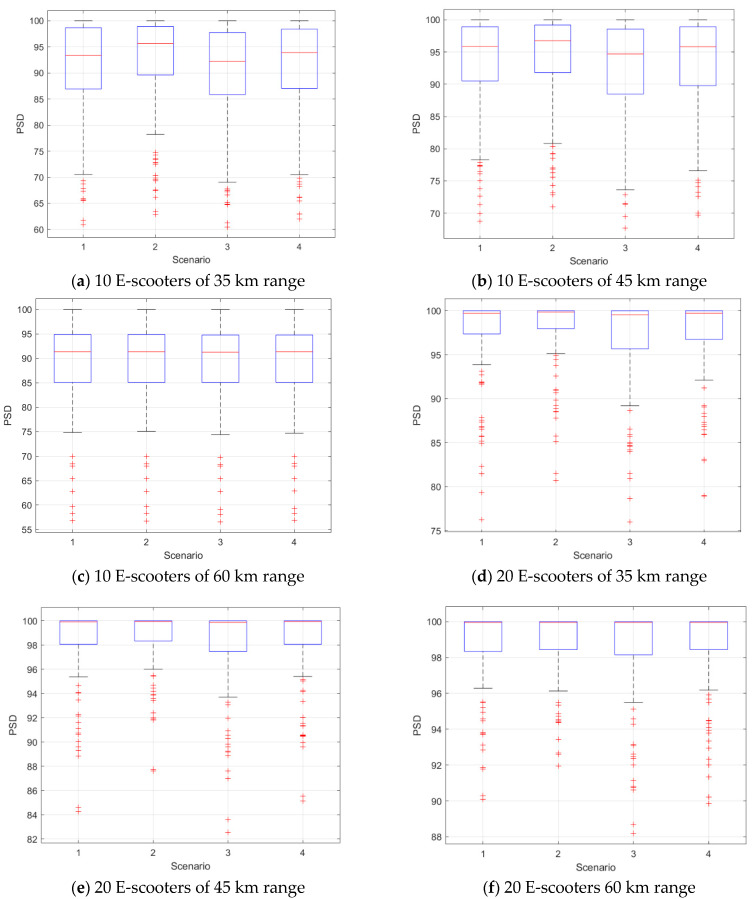
PSD results at different scenarios, number of e-scooters at each census tract, and battery ranges.

The PSD at different scenarios, different conditions of the e-scooter number and battery ranges were compared. Figure 5 shows box plots of the PSD versus the scenarios. There are minor PSD differences between the scenarios at the lower number of e-scooters per community area. The PSD increased as the used number of e-scooters in the model increased in each community area. Finally, we modelled the PSD results using a mixed-effect Gamma regression model as a function in the scenarios. Table 6 presents the *p*-values of the indicator variables corresponding to the scenarios. These *p*-values dictated that Scenarios 2 and 3 were different from Scenario 1 at all conditions.

## 5. Discussion and Future Work

The public interest in micro-mobility modes has recently been increasing rapidly, transforming the transportation systems in many cities. Micro-mobility offers an affordable and rapid commuting opportunity, thereby saving users from intolerable waiting and time wastage in congested areas. Micro-mobility is an important transportation mode that is needed for first and last-mile trips. During the COVID-19 pandemic, surveys have shown that more people have shifted to micro-mobility ride-sharing modes to avoid infection than other public transportation sectors like buses and trains. Another application of the proposed model is during pandemics after lockdowns have ended and after restrictions have been gradually eased. This model can serve as a solution to the issues arising after people return to normal life routines for economic recovery. However, precautions are required to minimize the chances of exposing areas to a second wave of the pandemic. Social distancing is one of the most important counter-measures against the spread of a pandemic, and it needs to be in effect for months after the peak. Social distancing is a challenging issue in cities where public transportation is the main commuting means. This model could be deployed in areas of a city to meet some demands of avoiding public transportation.

An ABM framework was built to control the proposed model using five different rules. We tested herein the model using two different datasets, Austin, TX and Chicago, Illinois. We used two criteria to evaluate the aggregated behaviour of the ABM model when changing the central agent rules over four different scenarios. We used two criteria: home index and percentage of satisfying demand. The simulation results show that the proposed model could contribute toward the solution of the first and last-mile problem.

A promising application of this novel model could be in a crowded city centre, where employees arrive at their offices early in the morning using e-scooters/e-bikes and stay until 5:00 PM. E-scooters/e-bikes could be offered for rent during the entire period they are at offices under the condition that at the end of the office hours, the e-scooter/e-bike is returned to the same point or a nearby point of their original location at the beginning of the day and has a residual charge enough for the owner to go back home.

In future work, we will investigate adding more complicated agent rules based on a mathematical model to improve the home index (HI). Moreover, we will develop an incentive system to encourage both suppliers and users to change home and drop-off locations. Finally, battery range is another interesting parameter that we need to study in the proposed model when we have heterogeneous battery ranges.

## Figures and Tables

**Table 1:** List of operators licensed to serve in Austin, TX.

Operator	E-Scooters	E-Bikes
Bird	4500	0
JUMP	2500	2000
Lime	5000	0
Lyft	2000	0
OjO	100	0
Skip	500	0
Spin	500	0
VeoRide	300	50
Total	15,400	2050

**Table 2:** Indicator variables used to encode the tested combination.

Scenarios	Rules	*X*_1_	*X*_2_	*X*_3_
Scenario 1	Rules 1 and 5	0	0	0
Scenario 2	Rules 2 and 5	1	0	0
Scenario 3	Rules 3 and 5	0	1	0
Scenario 4	Rules 4 and 5	0	0	1

**Table 3:** *p* values of the scenario’s indicator variables for the HI models.

E-Scooter Number and Battery Ranges	*X*_1_	*X*_2_	*X*_3_
50 E-scooters of 35 km range	0.67111	<0.0001	<0.0001
50 E-scooters of 45 km range	0.70196	<0.0001	<0.0001
50 E-scooters of 60 km range	0.64689	<0.0001	<0.0001
100 E-scooters of 35 km range	0.00899	<0.0001	<0.0001
100 E-scooters of 45 km range	0.00255	<0.0001	<0.0001
100 E-scooters of 60 km range	0.00304	<0.0001	<0.0001

**Table 4:** *p* values of the scenario’s indicator variables for the PSD models.

E-Scooter Number and Battery Ranges	*X*_1_	*X*_2_	*X*_3_
50 E-scooters of 35 km range	<0.0001	<0.0001	0.3976
50 E-scooters of 45 km range	0.0064019	<0.0001	<0.0001
50 E-scooters of 60 km range	0.1667	<0.0001	0.051345
100 E-scooters of 35 km range	0.0027793	<0.0001	<0.0001
100 E-scooters of 45 km range	0.34828	<0.0001	0.206
100 E-scooters of 60 km range	0.55138	0.0026561	0.26083

**Table 5:** *p* values of the scenario’s indicator variables for the HI models.

E-Scooter Number and Battery Ranges	*X*_1_	*X*_2_	*X*_3_
10 E-scooters of 35 km range	<0.0001	<0.0001	<0.0001
10 E-scooters of 45 km range	<0.0001	<0.0001	<0.0001
10 E-scooters of 60 km range	<0.0001	<0.0001	<0.0001
20 E-scooters of 35 km range	<0.0001	<0.0001	<0.0001
20 E-scooters of 45 km range	<0.0001	<0.0001	<0.0001
20 E-scooters of 60 km range	<0.0001	<0.0001	<0.0001

**Table 6:** *p*-values of the scenario’s indicator variables for the PSD models.

E-Scooter Number and Battery Ranges	*X*_1_	*X*_2_	*X*_3_
10 E-scooters of 35 km range	<0.0001	<0.0001	0.68926
10 E-scooters of 45 km range	<0.0001	<0.0001	0.40153
10 E-scooters of 60 km range	<0.0001	<0.0001	0.082923
20 E-scooters of 35 km range	<0.0001	<0.0001	0.91115
20 E-scooters of 45 km range	<0.0001	<0.0001	0.8515
20 E-scooters of 60 km range	0.0059286	<0.0001	0.55803

## Data Availability

Publicly available datasets were analyzed in this study. This data can be found here: [https://www.chicago.gov/scooters] (accessed on 6 July 2021) and [https://data.austintexas.gov/Transportation-and-Mobility/Shared-Micromobility-Vehicle-Trips/7d8e-dm7r] (accessed on 6 July 2021).
